# Supplementary material for: MRI Deep Learning-Based Solution for Alzheimer’s Disease Prediction
Source: J Pers Med. 2021 Sep 9;11(9):902. doi: 10.3390/jpm11090902 (PMC8466762; doi:10.3390/jpm11090902)
Supplement: Supplementary file 1 [file jpm-11-00902-s001.zip › jpm-1301122-Supplementary.pdf]

Table S1. Summary of cases excluded from the OASIS-2 dataset.

MRI ID	MRI ID	MRI ID
OAS2_0013_MR1	OAS2_0090_MR1	OAS2_0137_MR2
OAS2_0014_MR2	OAS2_0091_MR1	OAS2_0140_MR1
OAS2_0023_MR2	OAS2_0094_MR1	OAS2_0140_MR2
OAS2_0032_MR1	OAS2_0096_MR1	OAS2_0144_MR1
OAS2_0032_MR2	OAS2_0098_MR2	OAS2_0145_MR1
OAS2_0034_MR4	OAS2_0101_MR1	OAS2_0146_MR2
OAS2_0044_MR1	OAS2_0102_MR1	OAS2_0149_MR1
OAS2_0044_MR2	OAS2_0102_MR2	OAS2_0152_MR1
OAS2_0044_MR3	OAS2_0103_MR3	OAS2_0156_MR1
OAS2_0048_MR5	OAS2_0104_MR1	OAS2_0157_MR1
OAS2_0051_MR1	OAS2_0104_MR2	OAS2_0158_MR1
OAS2_0051_MR2	OAS2_0114_MR1	OAS2_0160_MR2
OAS2_0051_MR3	OAS2_0114_MR2	OAS2_0162_MR1
OAS2_0062_MR3	OAS2_0117_MR2	OAS2_0165_MR2
OAS2_0068_MR1	OAS2_0117_MR3	OAS2_0175_MR1
OAS2_0070_MR1	OAS2_0117_MR4	OAS2_0175_MR3
OAS2_0077_MR1	OAS2_0120_MR1	OAS2_0179_MR1
OAS2_0077_MR2	OAS2_0126_MR1	OAS2_0179_MR2
OAS2_0080_MR1	OAS2_0126_MR2	OAS2_081_MR1
OAS2_0081_MR1	OAS2_0127_MR3	OAS2_0182_MR2
OAS2_0085_MR2	OAS2_0127_MR4	OAS2_0185_MR1
OAS2_0087_MR1	OAS2_0134_MR2	OAS2_0185_MR3
OAS2_0088_MR1	OAS2_0135_MR1	
